# Aberrant TRPC1 expression reflects stromal cervical invasion, lymphovascular invasion, elevated FIGO stage, and poor survival in resectable endometrial carcinoma patients

**DOI:** 10.1002/jcla.24560

**Published:** 2022-06-26

**Authors:** Yi Wang, Chun Zhang

**Affiliations:** ^1^ Gynecology Department, The Central Hospital of Wuhan, Tongji Medical College Huazhong University of Science and Technology Wuhan China

**Keywords:** clinical features, endometrial carcinoma, multiple validations, survival, transient receptor potential channel 1

## Abstract

**Background:**

Transient receptor potential channel 1 (TRPC1) promotes tumor growth and metastasis in endometrial carcinoma (EC) cell lines, whereas its clinical role in EC management remains unclear. Therefore, this study aimed to investigate the association of TRPC1 protein expression with the clinical features and survival of EC patients, then was further validated by TRPC1 mRNA measurement and data from The Human Protein Atlas.

**Methods:**

TRPC1 protein expression in tumor tissues and normal endometria of 176 resectable EC patients was determined using immunohistochemistry. Besides, TRPC1 mRNA expression of partial patients (*n* = 80) was detected using RT‐qPCR. Additionally, survival data from The Human Protein Atlas (derived from The Cancer Genome Atlas [TCGA]) was analyzed.

**Results:**

TRPC1 protein expression was up‐regulated in tumor tissue compared with normal endometrium (*p* < 0.001). Up‐regulated TRPC1 protein expression was associated with stromal cervical invasion (*p* = 0.044), lymphovascular invasion (*p* = 0.032), and increased federation of gynecology and obstetrics (FIGO) stage (*p* = 0.005). Tumor TRPC1 protein high was linked with shortened accumulating disease‐free survival (DFS) (*p* = 0.009) and overall survival (OS) (*p* = 0.026), which were also confirmed by multivariate Cox's regression analysis (both *p* < 0.050). Further, TRPC1 mRNA validation disclosed that TRPC1 mRNA high was related to shortened accumulating DFS (*p* = 0.038) and exhibited a correlating trend with declined OS (lacked statistical significance) (*p* = 0.162). Meanwhile, survival analysis on the data from The Human Protein Atlas (derived from TCGA) also exhibited that TRPC1 mRNA high was correlated with reduced accumulating OS (*p* < 0.001).

**Conclusion:**

Our findings support TRPC1 as a prognostic biomarker in resectable EC patients.

## Graphical abstract

A total of 176 resectable endometrial carcinoma (EC) patients were recruited, then transient receptor potential channel 1 (TRPC1) protein and mRNA (of partial patients) in tumor tissues and normal endometria were determined. Additionally, survival data from The Human Protein Atlas (derived from The Cancer Genome Atlas [TCGA]) were analyzed. Subsequently, it was observed that TRPC1 protein was elevated in tumor tissue compared with normal endometrium (*p* < 0.001). Increased TRPC1 protein was associated with stromal cervical invasion (*p* = 0.044), lymphovascular invasion (*p* = 0.032), the elevated federation of gynecology and obstetrics (FIGO) stage (*p* = 0.005), shortened disease‐free survival (DFS) (*p* = 0.009), and overall survival (OS) (*p* = 0.026). Multivariate Cox's regression analysis confirmed its correlation with DFS and OS as well (both *p* < 0.050). Additionally, further TRPC1 mRNA validation disclosed that TRPC1 mRNA high was related to shortened DFS (*p* = 0.038). Meanwhile, survival analysis on the data from The Human Protein Atlas (derived from TCGA) also exhibited that TRPC1 mRNA high was linked with reduced OS (*p* < 0.001). Collectively, TRPC1 might serve as a prognostic biomarker in resectable EC patients.

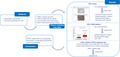

## 1 INTRODUCTION

Endometrial carcinoma (EC), representing 90% of uterine cancer, is one of the most common gynecologic malignant diseases worldwide, which can be divided into type I (estrogen‐dependent, accounting for 85% of EC cases) and type II (estrogen‐independent).^1^, ^2^, ^3^ In China, the incidence of EC is increasing annually with 63,400 new cases recently, which might due to the elevated prevalence of risk factors of EC obesity, hypertension, and diabetes (the main risk factors of EC).^4^, ^5^ Currently, the mainstay of treatment in most EC patients is total hysterectomy and bilateral salpingo‐oophorectomy; for patients who are not willing/suitable for surgery, conservative treatment including chemotherapy, radiation therapy, and hormonal therapy, is recommended.^6^, ^7^ However, despite the relatively delightful general prognosis of EC patients, a few patients still suffer from a high risk of recurrence and poor prognosis.^8^, ^9^, ^10^ Consequently, finding potential biomarkers reflecting the long‐term survival can provide some reference for the individualized adjuvant‐therapy selection of postoperative EC patients.

Transient receptor potential channel 1 (TRPC1) is a kind of voltage‐independent cation channel protein located on the cell membrane, which regulates calcium ions (Ca^2+^) influx and plays an oncogenic role in some solid cancers through activating calmodulin‐mediated phosphoinositide 3‐kinase/protein kinase B (PI3K/Akt) signaling axis, such as colorectal cancer, breast cancer, thyroid cancer, and pancreatic cancer.^11^, ^12^, ^13^, ^14^ For instance, one study identifies that TRPC1 overexpression promotes tumor growth and metastasis in human colorectal cancer cells.^13^ Another study finds that TRPC1 is positively related to the clinical stage and serves as an independent risk factor for metastasis in breast cancer patients.^14^ Notably, some studies observe that TRPC1 induces epithelial‐mesenchymal transition (EMT) in EC cell lines, which implies that TRPC1 might be involved in EC pathogenesis.^15^, ^16^ Nevertheless, the clinical role of TRPC1 in EC management has not been reported yet.

Hence, this study aimed to investigate the association of TRPC1 protein expression with the clinical features and survival of EC patients, then was further validated by TRPC1 mRNA measurement and data from The Human Protein Atlas derived from The Cancer Genome Atlas (TCGA) analysis.

## 2 MATERIALS AND METHODS

### 2.1 Patients

This study retrospectively analyzed 176 EC patients who underwent surgical resection between January 2015 and December 2020. Patients who met the following criteria were eligible for inclusion (a) were diagnosed as EC by histopathological examination; (b) were more than 18 years old; (c) received surgical resection; (d) had available specimens of tumor tissues for study use; (e) had adequate clinical data for study analysis. Exclusion criteria were (a) had a history of previous or concomitant cancer; (b) were pregnant or lactating. The study was permitted by Ethics Committee of The Central Hospital of Wuhan, Tongji Medical College, Huazhong University of Science and Technology.

### 2.2 Collection of data and specimens

Demographics, disease characteristics, and survival data of EC patients were collected. Disease‐free survival (DFS) and overall survival (OS) were imputed. The last follow‐up date was December 2021. A total of 176 formalin‐fixed paraffin‐embedded (FFPE) specimens of tumor tissue as well as 80 FFPE specimens of normal endometrium were collected from enrolled EC patients to examine TRPC1 protein expression. Besides, a total of 80 fresh tumor tissue specimens and 80 fresh normal endometrium specimens which were preserved with liquid nitrogen from enrolled EC patients were collected to detect TRPC1 mRNA expression.

### 2.3 Detection of TRPC1 protein expression

TRPC1 protein expression was examined by immunohistochemistry (IHC), and the experimentation of the IHC assay was in accord with a previous study.^17^ Briefly, the collected FFPE specimens were cut into slides. The slides were depolymerized in xylene, rehydrated, and then heated in a citric acid buffer at pH 6 to expose antigens. Sequentially. The slides were incubated with goat anti‐TRPC1 antibody (dilution 1:150; Abcam) as the primary antibody and rabbit anti‐goat IgG (H&L) (dilution 1:2000; Abcam) as the second antibody. After incubation, diaminobenzidine and hematoxylin were used for staining. The results of the IHC assay were semi‐quantitatively evaluated using a light microscope by 2 investigators who were blinded to the patient's clinical data according to the density and intensity of stained cells. The density was scored as 5 grades (score 0–4), and the intensity of stained cells was scored as 4 grades (score 0–3). The final score of the IHC assay was 12, which was a product of the density score and the intensity score. The final score of the IHC assay was a product of the density score and the intensity score, ranging from 0 to 12.

### 2.4 Detection of TRPC1 mRNA expression

TRPC1 mRNA expression was evaluated by reverse transcription‐quantitative polymerase chain reaction (RT‐qPCR). In brief, total RNA was extracted by TRIzol™ Reagent (Thermo Fisher Scientific); subsequently, reserve transcription was finished using PrimeScript™ RT reagent Kit (Takara). Then, the qPCR reaction was completed by SYBR® Premix DimerEraser™ (Takara). The relative expression was calculated using the 2^−ΔΔCt^ method, and GAPDH was used as the internal reference. The design of qPCR primer sequences referred to the previous study.^18^

### 2.5 Collection of TRPC1 RNA Fragments of Kilobase Million (FPKM) data

A total of 541 EC patients' RNA FPKM data were obtained from The Human Protein Atlas (derived from TCGA analysis, available at https://www.proteinatlas.org/ENSG00000144935‐TRPC1/pathology/endometrial+cancer) to further verify the correlation of TRPC1 expression with survival among EC patients.

### 2.6 Statistical analysis

For analysis, TRPC1 protein expression was classified based on the final IHC score in tumor tissue: high expression (>3); low expression (≤3). TRPC1 mRNA expression was classified based on the median value (3.040) in tumor tissue: high expression (≥3.040); low expression (<3.040). Patients' data were analyzed using SPSS 24.0 (IBM Corp.). Graphs were plotted using GraphPad Prism 7.01 (GraphPad Software Inc.). Paired‐samples *t* test or Wilcoxon signed‐rank test was used for the comparison of TRPC1 expression between tumor tissue and normal endometrium. The *t* test, one‐way analysis of variance (ANOVA) test, Wilcoxon rank‐sum test, or Kruskal–Wallis H rank‐sum test was used to compare tumor TRPC1 expression between or among patients with different characteristics. Spearman's rank correlation test was used for the correlation of tumor TRPC1 expression with clinical features. Kaplan–Meier curves and log‐rank test were used for DFS and OS assessment. Cox's proportional hazard regression model was used for prognostic analysis, and all potential factors were included in forward‐stepwise multivariate Cox's regression analysis for the screening of independence factors. *p* value <0.05 was considered significant.

## 3 RESULTS

### 3.1 Clinical characteristics

One hundred and seventy‐six EC patients with a mean age of 59.9 ± 9.8 years were recruited in this study, among which, 25 (14.2%) patients were pre‐menopause, while the other 151 (85.8%) patients were post‐menopause (Table 1). Regarding the histological subtype, 126 (71.6%), 13 (7.4%), 24 (13.6%), and 13 (7.4%) patients were respectively recognized as endometrioid carcinoma G1/G2, endometrioid carcinoma G3, serous EC, and clear cell EC. Additionally, 69 (39.2%), 40 (22.7%), and 46 (26.1%) patients presented myometrial invasion ≥50%, stromal cervical invasion, and lymphovascular invasion, correspondingly. As to the international federation of gynecology and obstetrics (FIGO) stage, 109 (61.9%), 21 (11.9%), 32 (18.2%), and 14 (8.0%) patients were assessed as stage I, II, III, and IV, respectively. The specific clinical characteristics of EC patients are displayed in Table 1.

**TABLE 1:** Characteristics of EC patients

Items	EC patients (*N* = 176)
Age (years), mean ± SD	59.9 ± 9.8
Menopausal status, *n* (%)	
Pre‐menopause	25 (14.2)
Post‐menopause	151 (85.8)
Diabetes, *n* (%)	
No	129 (73.3)
Yes	47 (26.7)
Hypertension, *n* (%)	
No	94 (53.4)
Yes	82 (46.6)
Histological subtype, *n* (%)	
Endometrioid carcinoma G1/G2	126 (71.6)
Endometrioid carcinoma G3	13 (7.4)
Serous endometrial carcinoma	24 (13.6)
Clear cell endometrial carcinoma	13 (7.4)
Myometrial invasion ≥50%, *n* (%)	
No	107 (60.8)
Yes	69 (39.2)
Cervical invasion, *n* (%)	
None or epithelial	136 (77.3)
Stromal	40 (22.7)
Lymphovascular invasion, *n* (%)	
No	130 (73.9)
Yes	46 (26.1)
FIGO stage, *n* (%)	
Stage I	109 (61.9)
Stage II	21 (11.9)
Stage III	32 (18.2)
Stage IV	14 (8.0)

Abbreviations: EC, endometrial carcinoma; SD, standard deviation; FIGO, International Federation of Gynecology and Obstetrics.

### 3.2 TRPC1 protein expression

**FIGURE 1:**
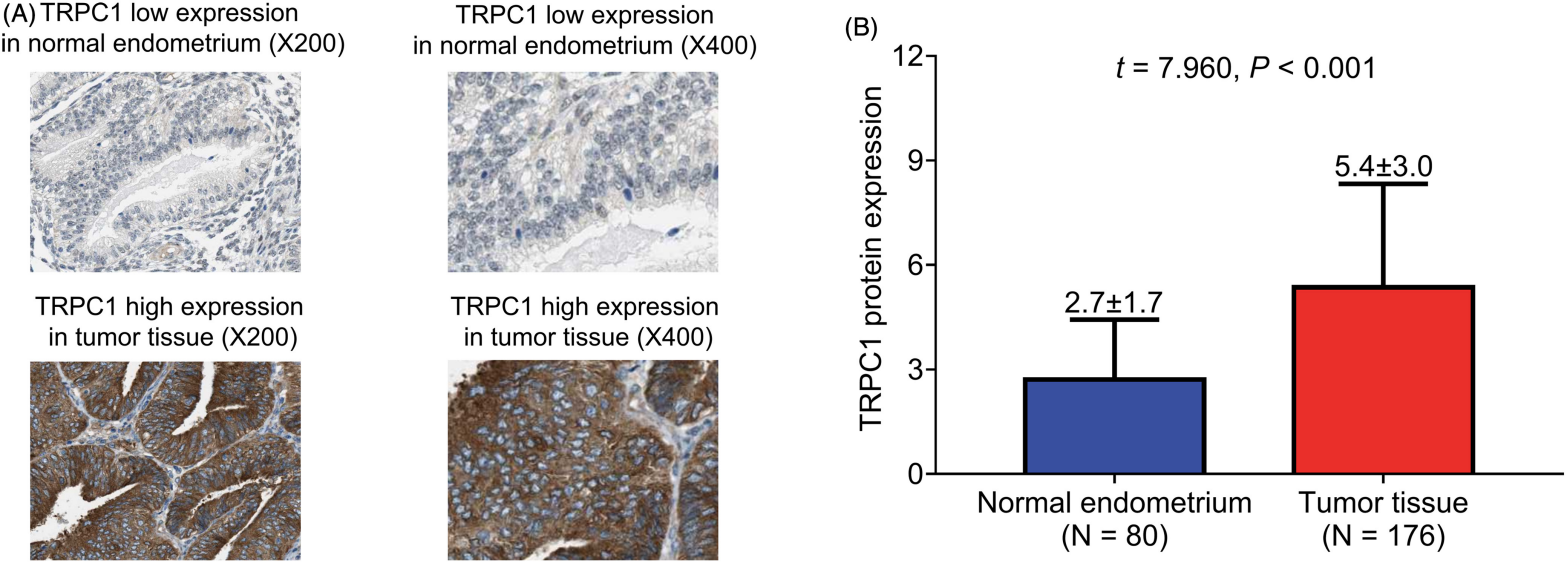
TRPC1 protein expression was elevated in tumor tissue compared with normal endometrium of EC patients. TRPC1 IHC stain examples (A) and TRPC1 protein expression reflected using IHC score (B) in normal endometrium and tumor tissue of EC patients

TRPC1 IHC stain examples in normal endometrium and tumor tissue of EC patients were exhibited (Figure 1A). TRPC1 protein expression was up‐regulated in tumor tissue compared with normal endometrium of EC patients (IHC score: 5.4 ± 3.0 vs. 2.7 ± 1.7, *p* < 0.001, Figure 1B).

### 3.3 Comparison of TRPC1 protein expression in patients with different clinical features

TRPC1 protein expression was increased in stromal cervical invasion patients compared to none or epithelial cervical invasion patients (IHC score: 6.2 ± 3.5 vs. 5.1 ± 2.8, *p* = 0.044). Besides, TRPC1 protein expression was elevated in lymphovascular invasion patients than that in non‐lymphovascular invasion patients (IHC score: 6.2 ± 3.3 vs. 5.1 ± 2.8, *p* = 0.032). TRPC1 protein expression was highest in stage IV patients (IHC score: 6.6 ± 3.4), followed by stage III (IHC score: 6.0 ± 3.2) and II (IHC score: 6.1 ± 3.5) patients, and lowest in stage I patients (IHC score: 4.9 ± 2.6) (*p* = 0.005) (Table 2).

**TABLE 2:** Correlation of tumor TRPC1 protein expression with clinical features

Items	TRPC1 protein expression			
	*n*	Mean ± SD	Statistic (*F*, *t*, *r*_*s*_)	*p* value
Age			1.469	0.144
<60 years	83	5.7 ± 3.0		
≥60 years	93	5.0 ± 2.9		
Menopausal status			−0.395	0.693
Pre‐menopause	25	5.1 ± 2.9		
Post‐menopause	151	5.4 ± 3.0		
Diabetes			−0.698	0.486
No	129	5.3 ± 2.9		
Yes	47	5.6 ± 3.2		
Hypertension			1.036	0.302
No	94	5.6 ± 2.8		
Yes	82	5.1 ± 3.1		
Histological subtype			0.968	0.409
Endometrioid carcinoma G1/G2	126	5.4 ± 2.9		
Endometrioid carcinoma G3	13	5.2 ± 2.4		
Serous endometrial carcinoma	24	4.6 ± 3.1		
Clear cell endometrial carcinoma	13	6.3 ± 3.8		
Myometrial invasion ≥50%			−0.782	0.436
No	107	5.2 ± 2.7		
Yes	69	5.6 ± 3.4		
Cervical invasion			−2.028	**0.044**
None or epithelial	136	5.1 ± 2.8		
Stromal	40	6.2 ± 3.5		
Lymphovascular invasion			−2.163	**0.032**
No	130	5.1 ± 2.8		
Yes	46	6.2 ± 3.3		
FIGO stage			0.208	**0.005**
Stage I	109	4.9 ± 2.6		
Stage II	21	6.1 ± 3.5		
Stage III	32	6.0 ± 3.2		
Stage IV	14	6.6 ± 3.4		

*Note*: Bold value represents statistical significance. Abbreviations: FIGO, International Federation of Gynecology and Obstetrics; SD, standard deviation; TRPC1, transient receptor potential canonical 1.

### 3.4 TRPC1 in patients with different menopausal statuses

The correlation of TRPC1 with the menopausal status of EC patients was relatively weak (*r*_*s*_ = −0.395, *p* = 0.693, Table 2). For further investigating the TRPC1 expression in patients with different menopausal statuses, a subgroup analysis was performed, which showed that TRPC1 protein expression was elevated in tumor tissue compared to normal endometrium of both pre‐menopause (IHC score: 5.1 ± 2.9 vs. 2.7 ± 0.9, *p* = 0.011, Supplementary Figure S1A) and post‐menopause (IHC score: 5.4 ± 3.0 vs. 2.7 ± 1.8, *p* < 0.001, Figure S1B) EC patients.

### 3.5 Correlation of TRPC1 protein expression with survival

**FIGURE 2:**
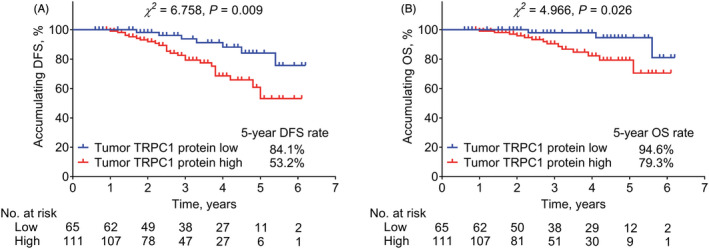
Tumor TRPC1 protein high linked with shortened DFS and OS in EC patients. Association of TRPC1 protein expression high with DFS (A) and OS (B) in EC patients

Tumor TRPC1 protein high was linked with shortened accumulating DFS (*P* = 0.009, Figure 2A) and OS (*p* = 0.026, Figure 2B) in EC patients. In detail, the 5‐year DFS and OS rates in TRPC1 protein high patients were correspondingly 53.2% and 79.3%; while in TRPC1 protein low patients, the 5‐year DFS and OS rates were 84.1% and 94.6%, respectively.

### 3.6 Factors related to DFS and OS

Tumor TRPC1 protein high (vs. low) was independently associated with reduced DFS in EC patients (hazard ratio [HR]: 2.963, *p* = 0.017). Serous EC (vs. endometrioid carcinoma G1/G2) (HR: 2.800, *p* = 0.025), clear cell EC (vs. endometrioid carcinoma G1/G2) (HR: 6.624, *p* < 0.001), and elevated FIGO stage (HR: 1.671, *p* = 0.001) were all independently correlated with declined DFS (Table 3).

**TABLE 3:** Factors related to DFS by Cox's proportional hazards regression analysis

Variables	*p* value	HR	95%CI	
			Lower	Upper
Univariate Cox's regression analysis				
Tumor TRPC1 protein (high vs. low)	**0.013**	2.919	1.251	6.811
Age (≥60 vs. <60 years)	**0.042**	2.185	1.030	4.633
Menopausal status (post‐menopause vs. pre‐menopause)	0.116	26.651	0.443	1.6 × 10^3^
Diabetes (yes vs. no)	0.164	1.680	0.810	3.487
Hypertension (yes vs. no)	0.200	1.580	0.784	3.184
Histological subtype				
Endometrioid carcinoma G1/G2	Ref.			
Endometrioid carcinoma G3	0.073	2.775	0.910	8.464
Serous endometrial carcinoma	**0.005**	3.498	1.465	8.351
Clear cell endometrial carcinoma	**0.001**	5.451	2.092	14.200
Myometrial invasion ≥50% (yes vs. no)	**0.022**	2.288	1.125	4.651
Cervical invasion (stromal vs. none or epithelial)	**0.001**	3.363	1.659	6.816
Lymphovascular invasion (yes vs. no)	**0.008**	2.566	1.279	5.150
FIGO stage	**<0.001**	1.722	1.287	2.304
Multivariate Cox's regression analysis				
Tumor TRPC1 protein (high vs. low)	**0.017**	2.963	1.216	7.218
Histological subtype				
Endometrioid carcinoma G1/G2	Ref.			
Endometrioid carcinoma G3	0.058	3.125	0.963	10.144
Serous endometrial carcinoma	**0.025**	2.800	1.135	6.911
Clear cell endometrial carcinoma	**<0.001**	6.624	2.497	17.570
FIGO stage	**0.001**	1.671	1.221	2.288

*Note*: Bold value represents statistical significance. Abbreviations: CI, confidence interval; DFS, disease‐free survival; FIGO, International Federation of Gynecology and Obstetrics; HR, hazard ratio; TRPC1, transient receptor potential canonical 1.

In terms of OS, tumor TRPC1 protein high (vs. low) was independently linked with shortened OS in EC patients (HR: 8.105, *p* = 0.007). Age ≥ 60 years (vs. <60 years) (HR: 5.478, *p* = 0.041), diabetes (vs. no) (HR: 3.949, *p* = 0.017), hypertension (vs. no) (HR: 3.392, *p* = 0.049), clear cell EC (vs. endometrioid carcinoma G1/G2) (HR: 8.563, *p* = 0.005), and stromal cervical invasion (vs. none or epithelial) (HR: 8.929, *p* < 0.001) were independently related to decreased OS (Table 4).

**TABLE 4:** Factors related to OS by Cox's proportional hazards regression analysis

Variables	*p* value	HR	95%CI	
			Lower	Upper
Univariate Cox's regression analysis				
Tumor TRPC1 protein (high vs. low)	**0.038**	3.795	1.080	13.335
Age (≥60 years vs. <60 years)	**0.010**	7.043	1.601	30.988
Menopausal status (post‐menopause vs. pre‐menopause)	0.264	26.376	0.085	8.2 × 10^3^
Diabetes (yes vs. no)	**0.003**	4.437	1.686	11.672
Hypertension (yes vs. no)	**0.048**	2.869	1.010	8.150
Histological subtype				
Endometrioid carcinoma G1/G2	Ref.			
Endometrioid carcinoma G3	0.982	<0.001	<0.001	NR
Serous endometrial carcinoma	0.104	2.704	0.814	8.987
Clear cell endometrial carcinoma	**<0.001**	8.141	2.649	25.020
Myometrial invasion ≥50% (yes vs. no)	0.131	2.112	0.801	5.571
Cervical invasion (stromal vs. none or epithelial)	**0.001**	5.073	1.910	13.471
Lymphovascular invasion (yes vs. no)	**0.013**	3.394	1.295	8.891
FIGO stage	**<0.001**	2.075	1.380	3.120
Multivariate Cox's regression analysis				
Tumor TRPC1 protein (high vs. low)	**0.007**	8.105	1.767	37.185
Age (≥60 years vs. <60 years)	**0.041**	5.478	1.070	28.049
Diabetes (yes vs. no)	**0.017**	3.949	1.274	12.242
Hypertension (yes vs. no)	**0.049**	3.392	1.007	11.428
Histological subtype				
Endometrioid carcinoma G1/G2	Ref.			
Endometrioid carcinoma G3	0.987	<0.001	<0.001	NR
Serous endometrial carcinoma	0.132	0.288	0.057	1.457
Clear cell endometrial carcinoma	**0.005**	8.563	1.940	37.790
Cervical invasion (stromal vs. none or epithelial)	**<0.001**	8.929	2.669	29.868

*Note*: Bold value represents statistical significance. Abbreviations: CI, confidence interval; FIGO, International Federation of Gynecology and Obstetrics; HR, hazard ratio; NR, not reach; OS, overall survival; TRPC1, transient receptor potential canonical 1.

### 3.7 Further validation of TRPC1's prognostic value

**FIGURE 3:**
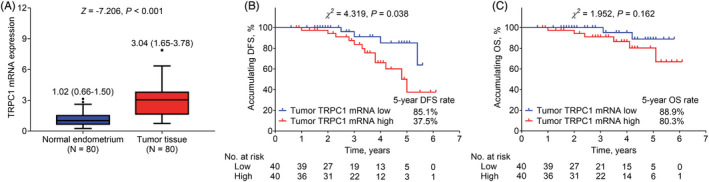
TRPC1 mRNA expression and its correlation with survival in EC patients. TRPC1 mRNA expression in normal endometrium and tumor tissue of EC patients (A). Association of TRPC1 mRNA expression high with DFS (B) and OS (C) in EC patients

For validating the prognostic value of TRPC1, this study further determined TRPC1 mRNA expression in a proportion of enrolled EC patients (n = 80). TRPC1 mRNA was increased in tumor tissue than that in normal endometrium of EC patients (median (interquartile range [IQR]): 3.04 (1.65–3.78) vs. 1.02 (0.66–1.50), *p* < 0.001, Figure 3A). Tumor TRPC1 mRNA high was related to shortened accumulating DFS (*p* = 0.038, Figure 3B) and exhibited a correlating trend with declined OS (lacked statistical significance) (*p* = 0.162, Figure 3C) in EC patients. Increased TRPC1 mRNA expression was associated with lymphovascular invasion (*p* = 0.039) and elevated FIGO stage (*p* = 0.049) in EC patients (Supplementary Table S1).

**FIGURE 4:**
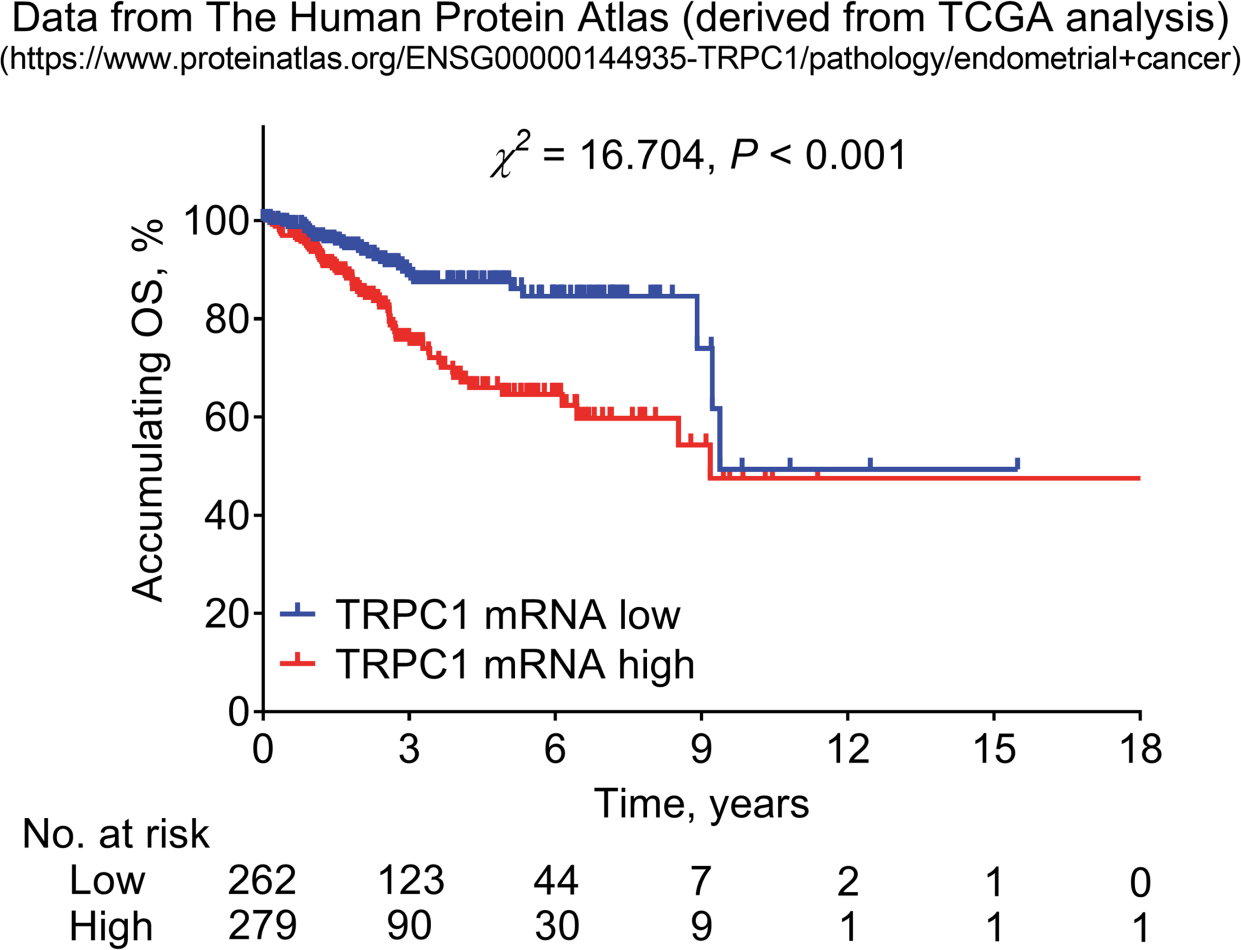
Further validation of TRPC1 prognostic value on the data from The Human Protein Atlas (derived from TCGA)

Besides, further survival analysis collected the data of 541 EC patients (with a mean age of 64.0 ± 11.1 years) from The Human Protein Atlas (derived from TCGA), which disclosed that TRPC1 mRNA high was correlated with reduced accumulating OS in EC patients (*p* < 0.001, Figure 4).

## 4 DISCUSSION

TRPC1, binding to stromal interaction molecule 1 (STIM1) and calcium release‐activated calcium channel protein 1 (ORAI1), works as a crucial store‐operated calcium ions (Ca^2+^) channel; meanwhile, its aberrant expression has been identified in several solid cancers.^14^, ^19^ For instance, one previous study shows that TRPC1 expression in breast cancer tissues is higher than that in normal breast tissues.^14^ Another study discloses that TRPC1 is up‐regulated in carcinoma tissue compared with para‐carcinoma tissue in non‐small cell lung cancer patients.^19^ Similarly, in this study, both TRPC1 protein and mRNA expressions were increased in tumor tissue compared with normal endometrium of EC patients. Possible reasons might be as follow: TRPC1 was positively linked with G protein‐coupled receptors (GPCRs), meanwhile, the latter was elevated in EC tissues compared with normal tissues according to the previous study.^20^, ^21^ As a result, TRPC1 was up‐regulated in tumor tissue than that in normal endometrium of EC patients. Besides, there was a mismatching of sample numbers between the TRPC1 protein and mRNA detection in the tumor and normal tissue, which could be explained by that TRPC1 protein expression was collected from FFPE specimens; while TRPC1 mRNA expression should be detected in fresh specimens preserved with liquid nitrogen, which was only available in partial patients.

Apart from the abnormal expression of TRPC1, the current study also found that TRPC1 high was related to stromal cervical invasion, lymphovascular invasion, and elevated FIGO stage in EC patients, which might be explained as follows: (1) TRPC1 elevated intracellular Ca^2+^ concentration via PI3K/Akt signaling pathways, then further facilitated the invasion of EC cells.^16^, ^22^ Therefore, TRPC1 high was correlated with stromal cervical invasion and lymphovascular invasion in EC patients. (2) FIGO stage was determined by tumor location, tumor size, lymph node metastasis, and pathologic metastasis; meanwhile, TRPC1 would facilitate cancer development and progression in EC.^15^, ^23^ Furthermore, in this study, up‐regulated TRPC1 was related to lymphovascular invasion in EC patients. Herein, TRPC1 high was linked with increased FIGO stage in EC patients.

Regarding the relationship between TRPC1 and survival in solid cancers, one previous study exhibits that the abnormal expression of TRPC1 is related to poor DFS and OS in esophageal squamous cell carcinoma patients.^24^ Another study also recognizes that up‐regulated TRPC1 is correlated with poor DFS, but is not linked with OS in non‐small cell lung cancer patients.^19^ In the current study, tumor TRPC1 protein high was linked with reduced accumulating DFS and OS in EC patients; meanwhile, the TRPC1 mRNA validation and survival analysis about the data from The Human Protein Atlas (derived from TCGA) obtained similar findings. Probable explanations were as follows: (1) As discussed above, up‐regulated TRPC1 exacerbated cancer invasion via regulating the mitogen‐activated protein kinase (MAPK) pathway.^25^ (2) TRPC1 promoted EMT in endometrial epithelial cells and underlying endometrial stromal cells; meanwhile, EMT accelerated cancer migration.^15^, ^26^ Combining the above two aspects, TRPC1 high was correlated with shortened DFS and OS in EC patients. Currently, surgical FIGO stage, myometrial invasion, histological type, and differentiation grades are the most widely used prognostic factors of EC; meanwhile, the combined application of TRPC1 might enhance their prognostic value.^27^, ^28^ Furthermore, the different findings between this study and the TCGA database could be explained by that the follow‐up duration of the current study was relatively shorter than that in the TCGA database, while the survival significance was mainly presented after 8 years.

Some limitations occurred in the current study: (1) This was a retrospective study, which might cause patients' selection bias. (2) The current study only enrolled surgically resectable patients and most of the recruited patients were at FIGO stage I; while the TRPC1 expression in advanced EC patients deserved further validation. (3) Additional in vivo and in vitro studies were necessary to explore whether TRPC1 could be recognized as a good therapeutic target for EC.

In conclusion, TRPC1 serves as a prognostic biomarker, whose overexpression reflects stromal cervical invasion, lymphovascular invasion, elevated FIGO stage, and poor survival in resectable EC patients.

## CONFLICT OF INTEREST

The authors declare that they have no conflict of interest.

## Supporting information


Figure S1

Table S1

## Data Availability

The datasets used and/or analyzed during the current study are available from the corresponding author on reasonable request.
