# Supplementary figures and images for: Aberrant TRPC1 expression reflects stromal cervical invasion, lymphovascular invasion, elevated FIGO stage, and poor survival in resectable endometrial carcinoma patients
Source: J Clin Lab Anal. 2022 Jun 26;36(8):e24560. doi: 10.1002/jcla.24560 (PMC9396166; doi:10.1002/jcla.24560)

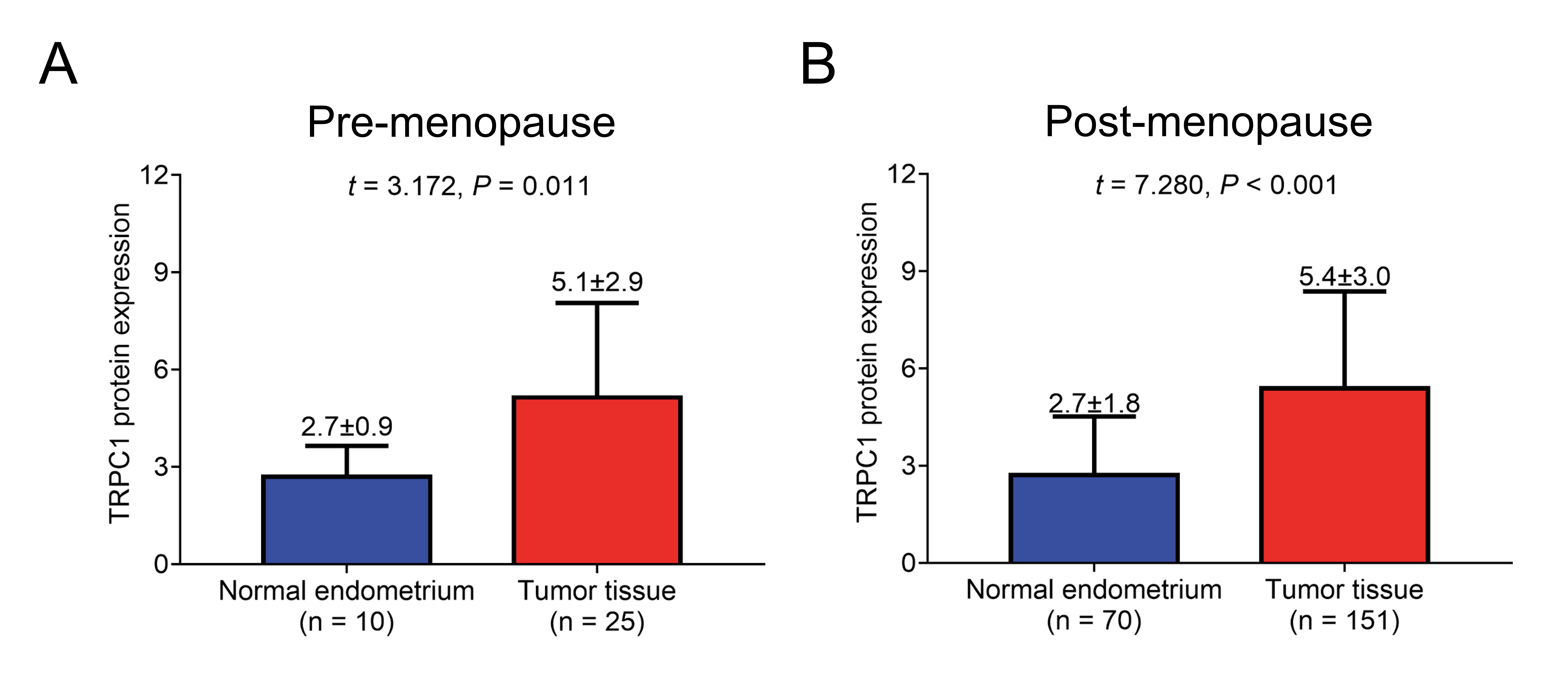

Supplement: Supplementary file 1 — Figure S1 [file JCLA-36-e24560-s001.tif]
