# Supplementary material for: Aberrant TRPC1 expression reflects stromal cervical invasion, lymphovascular invasion, elevated FIGO stage, and poor survival in resectable endometrial carcinoma patients
Source: J Clin Lab Anal. 2022 Jun 26;36(8):e24560. doi: 10.1002/jcla.24560 (PMC9396166; doi:10.1002/jcla.24560)
Supplement: Supplementary file 2 — Table S1 [file JCLA-36-e24560-s002.docx]

**Supplementary table 1.** Correlation of tumor TRPC1 mRNA expression with clinical features.

| Items | TRPC1 mRNA expression | | | | |
| --- | --- | --- | --- | --- | --- |
|  | n | Median (IQR) | Statistic (*H*, *Z*, *r_s_*) | | *P* value |
| Age |  |  | -0.101 | 0.919 | |
| <60 years | 41 | 2.91 (1.95-3.71) |  |  | |
| ≥60 years | 39 | 3.17 (1.45-3.94) |  |  | |
| Menopausal status |  |  | -0.502 | 0.616 | |
| Pre-menopause | 10 | 2.62 (1.35-3.67) |  |  | |
| Post-menopause | 70 | 3.04 (1.77-3.80) |  |  | |
| Diabetes |  |  | -0.544 | 0.586 | |
| No | 54 | 3.19 (1.68-3.83) |  |  | |
| Yes | 26 | 2.51 (1.54-3.77) |  |  | |
| Hypertension |  |  | -0.857 | 0.392 | |
| No | 46 | 3.43 (1.86-3.81) |  |  | |
| Yes | 34 | 2.66 (1.48-3.77) |  |  | |
| Histological subtype |  |  | 1.069 | 0.785 | |
| Endometrioid carcinoma G1/G2 | 53 | 2.82 (1.62-3.74) |  |  | |
| Endometrioid carcinoma G3 | 5 | 3.52 (2.54-3.82) |  |  | |
| Serous endometrial carcinoma | 13 | 2.42 (1.48-3.99) |  |  | |
| Clear cell endometrial carcinoma | 9 | 3.17 (1.47-5.15) |  |  | |
| Myometrial invasion ≥50% |  |  | -0.560 | 0.576 | |
| No | 53 | 2.79 (1.52-3.75) |  |  | |
| Yes | 27 | 3.34 (1.69-3.85) |  |  | |
| Cervical invasion |  |  | -0.968 | 0.333 | |
| None or epithelial | 64 | 2.81 (1.46-3.78) |  |  | |
| Stromal | 16 | 3.36 (1.96-4.88) |  |  | |
| Lymphovascular invasion |  |  | -2.062 | **0.039** | |
| No | 57 | 2.58 (1.42-3.74) |  |  | |
| Yes | 23 | 3.41 (2.21-3.94) |  |  | |
| FIGO stage |  |  | 0.221 | **0.049** | |
| Stage I | 52 | 2.66 (1.34-3.76) |  |  | |
| Stage II | 5 | 2.56 (1.78-3.23) |  |  | |
| Stage III | 15 | 3.41 (2.21-3.84) |  |  | |
| Stage IV | 8 | 4.35 (2.40-6.21) |  |  | |

TRPC1, transient receptor potential canonical 1; IQR, interquartile range; FIGO, International Federation of Gynecology and Obstetrics; bold value represents statistical significance.
